# Irish Nurses' League

**Published:** 1917-11-24

**Authors:** 


### Irish Nurses' League.

We are now in the happy position of having three organisations in Ireland endeavouring to solve the nursing problem, for at a meeting of nurses recently held in Dublin it was decided to establish the Irish Trained Nurses' League. The pernicious effect of Dr. Kirkpatrick's pamphlet is in strong evidence here, for in addition to setting up State Registration as a desirable goal the League aims at making it illegal for untrained persons to practise nursing for gain. It is deeply to be deplored that faction should thus be deliberately encouraged by persons who ought to know better. There is not the smallest reason why all Irish trained nurses should not enrol under a common banner, for, so far as they are concerned, their aims are identical, excepting in a case like this where a section are seeking after the impossible. They think, doubtless, that they are going one better than the rest of the nursing world, but in reality they are weakening the cause. And it is hard to blame them when men well known in the medical profession promulgate such a fallacy as that nursing can be made by law a protected profession. The Midwives Bill, which I have just been discussing, clearly demonstrates the attitude of the Legislature in this matter. Women are by its terms forbidden from attending women in childbirth for gain otherwise than under the direction of a registered medical practitioner. It ought to be obvious to every person of intelligence who gives the subject a moment's reflection that where a doctor is in attendance any person, male or female, trained or untrained, may perform the duty of nursing, no matter from what the patient may suffer. Institutions belonging to the State or to the public may make their own rules: public opinion may influence these rules, as, for example, in the case of hospitals, but the law will not interfere. Hence the need for active and widespread propaganda. Nurses require to be educated in matters appertaining to their welfare. At present there is far too much of the "Codlin's the friend—not Short" gospel in vogue. There are Codlins and Shorts here, there, and everywhere, and the minimum of attention is paid to principles. One of Pope's incisive epigrams is that "party is the madness of many for the gain of a few," and this applies with special force to the unorganised profession of nursing. What is wanted is a clearly defined programme which every trained nurse can without effort comprehend —a programme showing that her interests alone are concerned, and that these interests cannot be fully realised without union.

It is eminently desirable that there should be in this kingdom, and throughout the British Dominions, a solid unified body of trained nurses, with fixity of purpose and a determination to achieve the best possible during service and after. I am persuaded, moreover, that such a union would receive the warm support of an appreciative and grateful public. The faction-monger is the nurses' *bête noire*. IERNE.

